# Trim45 is essential to the development of the diencephalon and eye in zebrafish embryos

**DOI:** 10.1080/19768354.2020.1751281

**Published:** 2020-04-22

**Authors:** Seoyeon Choe, Tae-Lin Huh, Myungchull Rhee

**Affiliations:** a Department of Biological Sciences, College of Biosciences and Biotechnology, Brain Korea 21 Plus, Chungnam National University, Daejeon, South Korea; b School of Life Sciences and Biotechnology, College of Natural Sciences, Kyungpook National University, Daegu, South Korea

**Keywords:** E3 ligases, *trim45*, diencephalon, eye, zebrafish embryos

## Abstract

Trim45 is one of the RING (really interesting new gene) finger containing E3 ligase, which belongs to TRIM (Tripartite motif) protein family. Its molecular biological functions have been well characterized but not in light of developmental aspects. Here, we are reporting its expression patterns and developmental functions in zebrafish embryos. First, maternal transcripts of *trim45* were found at one cell stage while its zygotic messages appeared at 30% epiboly. *trim45* transcripts were restricted to the optical tectum, hypothalamus, hindbrain, and pharyngeal endoderm at 24 hpf (hour post-fertilization), and further to the retinal ganglion cell layer and cranial ganglion at 36 hpf. Second, ectopic expression of *trim45* by injecting its mRNAs into embryos at one cell stage caused significant expansion of the diencephalon and eye fields at 24 hpf. In contrast, knock-down of *trim45* with anti-sense *trim45* morpholinos reduced the size of the two tissues at 24 hpf. Finally, the spatial distribution of the transcripts from *olig2* and *rx1*/*rx3*, markers for the midbrain and eye respectively, were significantly decreased in the thalamus and eye fields respectively at 24 hpf. Based upon these observations, we proposed possible roles of Trim45 in the development of the diencephalon and eye in zebrafish embryos.

## Introduction

Ubiquitylation of proteins occurs in diverse forms to regulate wide ranges of cellular functions. Mono-ubiquitination, which covalently links a single ubiquitin to a protein, has a role in protein processes, such as trafficking, interaction, and degradation while multi-ubiquitination on an identical target protein is generally involved in endocytosis (Rape 2018). In the process of ubiquitylation, E3 ubiquitin ligases are associated with direct attachment of ubiquitin to their target proteins so that they give specificity for their target proteins (Venuto and Merla 2019). More than 600 members of E3 ligases have been identified in mammals whereas they are classified into four families depending upon types of the conserved domains, such as E6-AP C-terminus (HECT), really interesting new gene (RING), U-box, and PHD domain (Nakayama and Nakayama 2006). Tripartite motif (TRIM)-containing family is one of the E3 ubiquitin ligase families with RING domain. TRIM proteins have three distinct domains including RING finger, B-box, and coiled-coil domain. The RING motif has been evolutionary conserved and considered to have a role as a protein–protein interaction (Meroni and Diez-Roux 2005). TRIM45 belongs to TRIM family, containing a RING finger, two B-boxes, and a coiled-coil domain as well as a distinctive filamin-type immunoglobulin (IG-FLMN) domain at the C-terminus (Wang et al. 2004).

*TRIM45* is abundantly expressed in the brain of human embryo (Wang et al. 2004). In COS-7 cell line, overexpression of TRIM45 negatively regulates mitogen-activated protein kinase (MAPK)-mediated signaling pathway by inhibiting the transcription of ETS-like transcription factor 1 (Elk-1) and a transcriptional activator protein 1 (AP-1) (Wang et al. 2004). TRIM45 overexpression suppressed cell growth by negatively regulating the tumor necrosis factor α (TNFα) in human HeLa and HEK293T cell line (Shibata et al. 2012). TRIM45 directly interacts with the receptor for activated C-kinase (RACK1) to negatively regulate MAPK signaling by inhibiting RACK1/protein kinase C (PKC) complex formation (Sato et al. 2015) while it directly interacts with p53 by its FLMN domain to conjugate K63-linked polyubiquitination chain to stabilize and activate p53 in glioblastoma cell lines (Zhang et al. 2017). Although the molecular and cellular functions of TRIM45 have been studied in detail, its developmental functions have not been examined. It has been reported that proper development of the central nervous system in vertebrate embryogenesis involves various types of E3 ligases. An E3 ligase, Rnf152 plays essential roles in brain patterning via regulation of *neuroD* expression and Delta-Notch signaling in the zebrafish embryos (Kumar et al. 2017). Peli1b, a member of E3 ligase governs the brain patterning via ERK signaling pathways in zebraﬁsh embryos (Kumar et al. 2019). A ring zinc finger E3 ligase, March5 governs the convergence and extension movement for organization of the telencephalon and diencephalon in zebrafish embryos (Jung et al. 2020). Znf76 targets the TATA-binding protein as a transcriptional repressor regulates development of the eyes, midbrain, MHB, and hindbrain in zebrafish embryos (Jung et al. 2019).

It is thus of great intrigue to examine embryological roles of *trim45* in vertebrate embryogenesis using the zebrafish animal model. We performed the spatio-temporal expression analysis and using whole-mount *in situ* hybridization (WISH) and functional analysis of *trim45* by overexpression and knock-down of *trim45*. *trim45* was specifically expressed in the brain region and knock-down of *trim45* induced significant morphological defects in the midbrain and eyes as well as size reduction of the hypothalamus accompanying decrease in transcripts levels of retinal homeobox gene 1 (*rx1*) and 3 (*rx3*) (Chuang and Raymond 2001; Loosli et al. 2003). We discussed putative biological functions of Trim45 in the process of the corresponding tissues during the zebrafish embryogenesis.

## Materials and methods

### Zebrafish maintenance

Wild-type AB strain zebrafish were maintained at 28.5°C with 14 h light/10 h dark cycles. Embryos were obtained by natural mating, reared in 1X embryo medium at 28.5°C, and treated with 0.002% phenylthiourea (PTU) from 10 hpf. Embryos were staged according to Kimmel description (Kimmel et al. 1995).

### Preparation of probe

*trim45*-specific DNA sequences starting at 301 bp of zebrafish *trim45* ORF sequence through 524 bp were amplified by PCR using following primers: *trim45*-forward primer – 5′-ACTGATCACCTTGCGCTGGA-3′ and *trim45*-reverse primer – 5′-GGCCTTGTAAGACGACCCTGA-3′. pGEM-T-easy-*trim45* was constructed with the amplified *trim45* cDNA (224 bp). The construct was linearized with *Sac*I, and used as a template for synthesis of digoxigenin labeled-*trim45* anti-sense mRNAs.

### Whole-mount *in situ* hybridization

Whole-mount *in situ* hybridization was performed according to the protocol described by Thisse and Thisse (2008).

### Synthesis and overexpression of *trim45* mRNAs

Open reading frame (ORF) sequences of zebrafish *trim45* were synthesized using following primers: *trim45*-ORF-forward primer – 5′-GCGGATCCATGTCACTTTGTAAAGAGAAGGG-3′ and *trim45*-ORF-reverse primer – 5′-ATATGATATCGAGCTCCACAGTGCGGAGGT-3′. The synthesized DNA fragment was cloned into pGEM-T easy vector and digested by *Bam*HI and *Eco*RV restriction enzymes. The isolated cDNAs containing trim45 mRNA were ligated with pcGlobin2 vector (Ro et al. 2004) which was linearized by *Bam*HI and *Eco*RV. The *trim45* ORF + pcGLobin2 construct was linearized by *Xba*I enzyme and subjected to *in vitro* transcription using the mMessage mMachine T7 kit (Ambion). Approximately, 50–100 ng of *trim45* mRNA (200 ng–400 ng/μl) was injected into embryos at one cell stage using a micromanipulator (Narishige MN-151).

### Morpholino(MO)-mediated knock-down

The morpholino oligonucleotides (5′-AGTCAGTGATTAAACTAACCTGTGG-3′) contain anti-sense sequences of *trim45* and were microinjected into the yolk of embryos at one cell stage for knock-down of *trims45*. Embryos were observed under the stereomicroscope (Leica MZ16).

## Results

### Identification and isolation of zebrafish *trim45*

**Figure 1.**
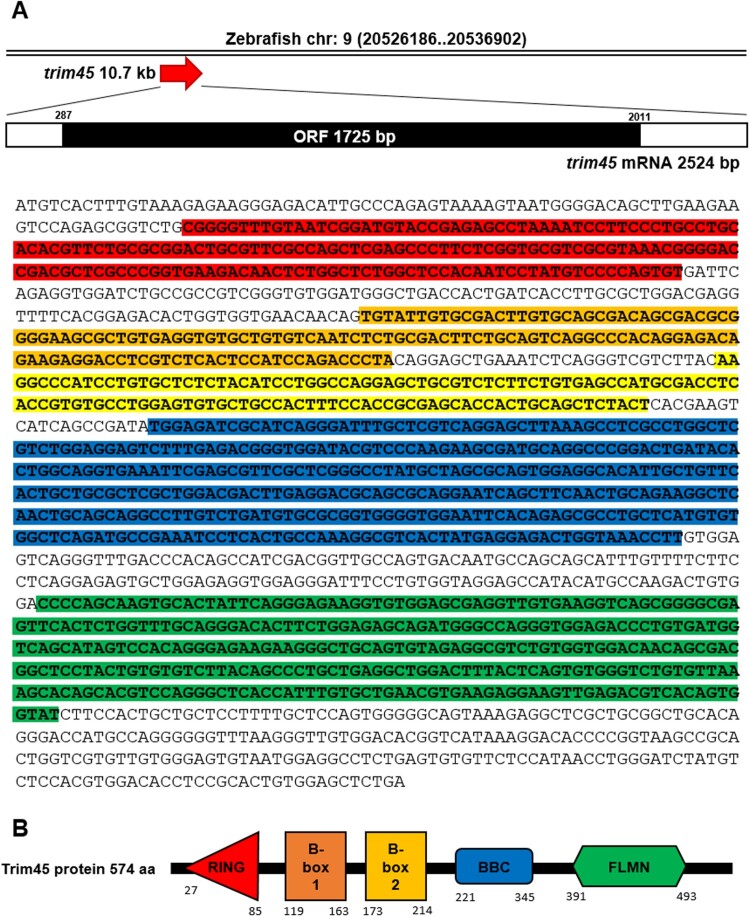
Structural characterization of zebrafish *trim45*. (A) Zebrafish trim45 is present on chromosome 9 and spans 10717 bp on the genome. trim45 translates 1725 bp of ORF which encodes 574 aa of Trim45 protein. Represented sequences are *trim45* ORF and each colored sequence encodes the corresponding colored protein domain below. (B) The domain structure of Trim45. Shown are the schematic diagram of RING (amino acids 27–85), B-box1 (amino acids 119–163), B-box2 (amino acids 173–214), B-box C-terminal domain (BBC) (amino acids 221–345), and IG-FLMN (amino acids 391–493) domain of Trim45 protein.

To identify zebrafish *trim45*, the NCBI database was used as a reference. Zebrafish *trim45* (Gene ID – 558117) is mapped to chromosome 9 and located in NC_007120.7 (20526186..20536902) which spans about 10.7 kb of nucleotides. *trim45* transcribes 2524 bp of mRNA and the ORF sequences from 287 bp to 2011 bp encodes 574 amino acids (Figure 1(A)). Trim45 protein structure was analyzed by protein database SMART (Simple Modular Architecture Tool) and 5 putative domains were found. Trim45 consists of the RING finger domain (175 a. a), B-box1 domain (44 a. a), B-box2 domain (41 a. a), B-box C-terminal domain (124 a. a), and IG-FLMN domain (103 a. a) (Figure 1(B)). To compare the homology with other vertebrates, protein sequences of human (*H. sapiens*), mouse (*M. musculus*), and zebrafish were aligned by the Jalview program for multiple sequence alignment editing. It shows that zebrafish Trim45 shares homology with human and mouse by 49% and 50%, respectively (data not shown).

### Spatio-temporal expression patterns of *trim45* in zebrafish embryos

TRIM45 has been implicated to be associated with various signaling pathways to modulate cellular processes. It negatively regulates the mitogen-activated protein kinase (MAPK)-mediated signaling pathway (Loosli et al. 2003) and the tumor necrosis factor α (TNFα) to suppress cell growth in a mammalian cell line (Shibata et al. 2012). It also directly interacts with p53 to conjugate the K63-linked polyubiquitination chain to stabilize and activate p53 in glioblastoma cell lines (Zhang et al. 2017). Although biological functions of TRIM45 have been characterized at the molecular biological level, its roles in embryogenesis remain to be further defined.

**Figure 2.**
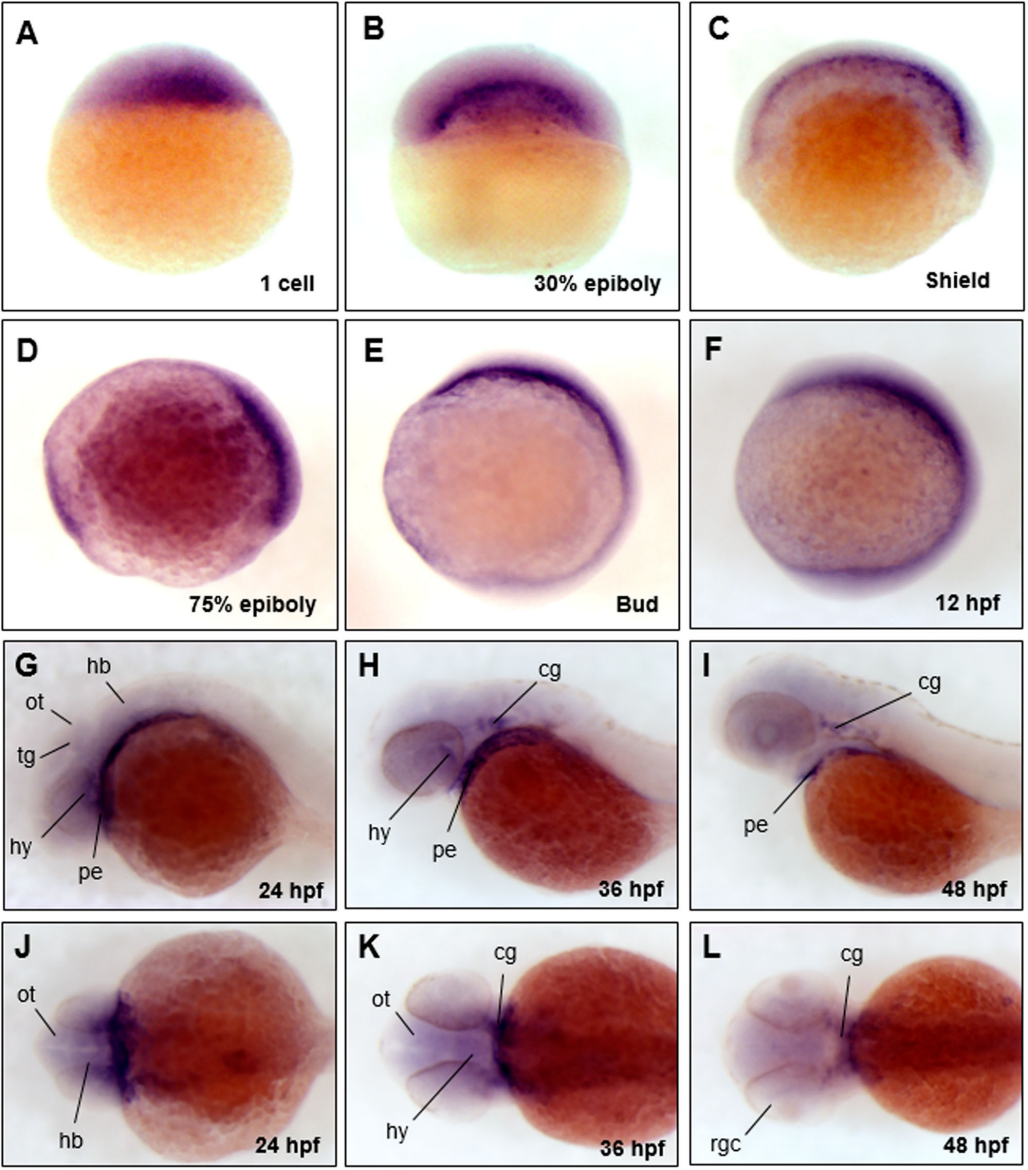
The spatio-temporal expression patterns of *trim45* in early embryonic stages of zebrafish. Whole mount *in situ* hybridization (WISH) was performed using digoxygenin-labeled *trim45* RNA probe at one cell, 30% epiboly, shield, 75% epiboly, bud, 12, 24, 36, 48 hpf stages. *trim45* had maternal messages from one cell stage (A). From 30% epiboly stage (B) to shield stage (C), expression was restricted to the yolk syncytial layer (YSL). At 75% epiboly (D), *trim45* was mainly expressed in dorsal mesoderm. At bud and 12 hpf stage (E and F), *trim45* was strongly expressed in the head region of the anterior pole. At 24 hpf stage, *trim45* was expressed in pharyngeal endoderm, tegmentum, optic tectum, and hindbrain (G and J). At 36 hpf stage, *trim45* was expressed in hypothalamus, pharyngeal endoderm, and cranial ganglion (H and K). At 48 hpf stage, *trim45* was expressed in pharyngeal endoderm, cranial ganglion, and retinal ganglion cell layer (I and L). Embryo orientations: lateral views (A–I) and dorsal views (J–L). Black lines point to various anatomical structures. pe, pharyngeal endoderm; ot, optic tectum; tg, tegmentum; te, telencephalon; mb, midbrain; hb, hindbrain; hy, hypothalamus; rgc, retinal ganglion cell layer; cg, cranial ganglion.

As an initial effort to evaluate the functional roles of Trim45, we examined spatio-temporal expression patterns of *trim45*. We conducted whole-mount *in situ* hybridization (WISH) using a *trim45*-specific anti-sense probe in zebrafish embryos at various developmental stages. *trim45* was maternally expressed and evenly distributed in the embryos at one cell (Figure 2(A)) and high stage (data not shown). Its zygotic transcripts appeared in the yolk syncytial layer (YSL) at 30% epiboly (Figure 2(B)) and became more restricted to the shield area at shield stages (Figure 2(C)). *trim45* transcripts became accumulated in the dorsal mesoderm but reduced in the ventral region of the embryos at 75% epiboly (Figure 2(D)). They were restricted to the anterior tissues of embryos at the bud stage and 12 hpf (Figure 2(E, F)). *trim45* messages were found specifically in the tegmentum, optic tectum, hypothalamus, and hindbrain region, and yet heavily accumulated in the pharyngeal endoderm at 24 hpf (Figure 2(G, J)). They were further concentrated in the cranial ganglion at 36 hpf (Figure 2H, K) as well as in the retinal ganglion cell layer at 48 hpf (Figure 2(I, L)). It is thus of interest to examine if the dynamic spatio-temporal expression patterns of *trim45* in the brain region is associated with CNS development along the embryonic development.

### Functional analysis of *trim45* in zebrafish embryogenesis

**Figure 3.**
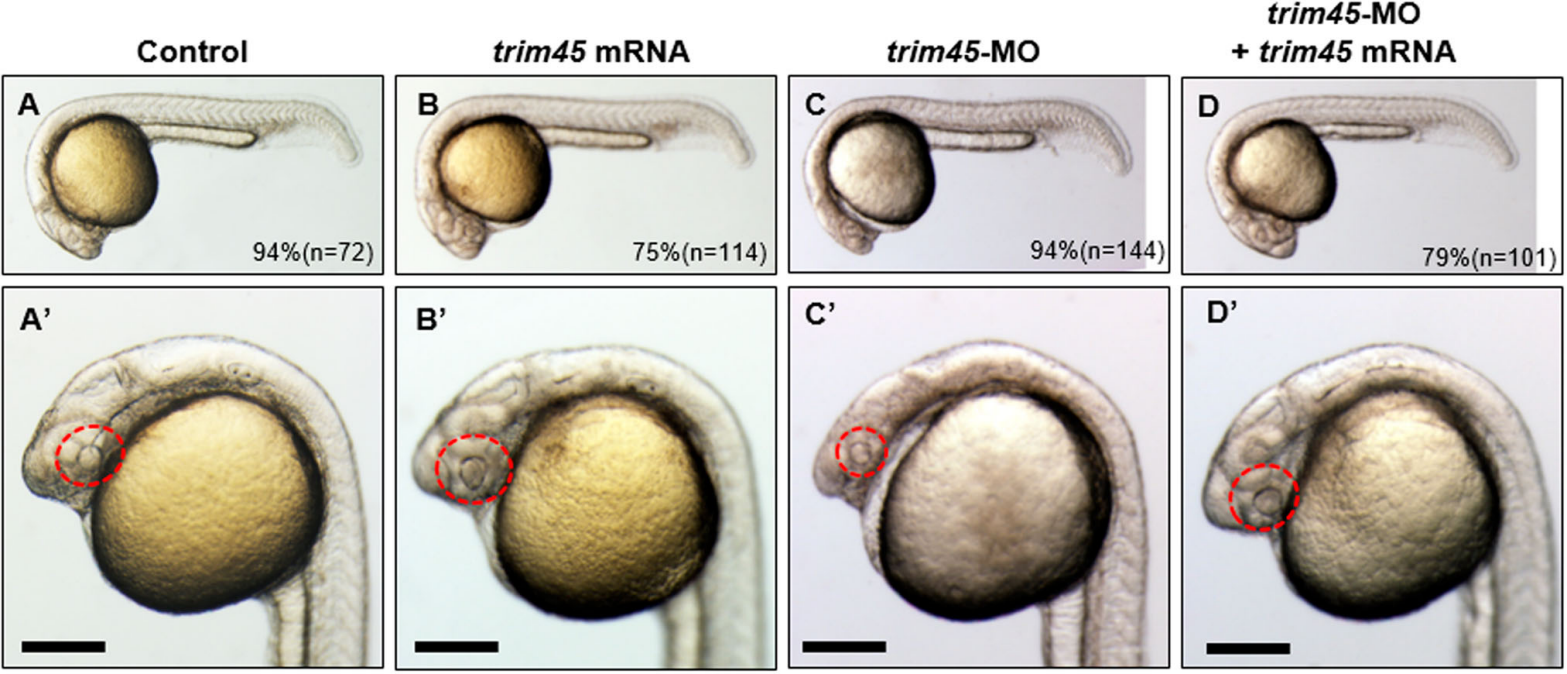
Phenotypes of the embryos from the overexpression or knock-down of *trim45* in zebrafish embryos. Bright-field images of live embryos observed at 24 hpf stage. Un-injected embryos were used as a control (A and A’). *trim45* mRNA (50 pg), *trim45*-MO (5 ng), and a mixture of *trim45*-MO (10 ng) and *trim45* mRNA (10 pg) were injected into one cell stage zebrafish embryos respectively. Overexpression of *trim45* showed a slight size expansion of midbrain and eyes (B and B’). *trim45* morphants showed shrinkage in the size of midbrain and eyes (C and C’). About 79% of embryos, which were co-injected with *trim45* mRNA, restored the wild-type phenotype (D and D’). Red dots – eye. Scale bar – 200 μm.

TRIM45 suppresses cell growth via negative regulation of TNFα when it is overexpressed in human HeLa and HEK293T cell line (Shibata et al. 2012). We thus examined if *trim45* contributes to brain development via regulation of cell growth in the tissues where *trim45* transcripts were present in the zebrafish embryos. Overexpression of trim45 was conducted by injecting 50 pg of *trim45* mRNAs into an embryo at one cell stage. Embryos at 24 hpf overexpressing *trim45* showed significant expansion of the midbrain and eyes (Figure 3(B, B’)). In contrast, knock-down of *trim45* was induced with 5 ng of anti-sense *trim45* morpholinos (*trim45* MOs) injected into an embryo (*trim45* morphants) at one cell stage. *trim45* morphants demonstrated remarkably reduced size in the midbrain and eyes (Figure 3(C, C’)) which was obviously opposite phenotypes found in the embryos overexpressing *trim45* (Figure 3(B, B’)). Taken together, all the phenotypic changes in the midbrain and eye upon overexpression and knock-down of *trim45* clearly implicate that Trim45 might be involved in the development of the two tissues in the zebrafish embryogenesis. To test this possibility, we conducted rescue experiments by co-injection of *trim45* mRNAs together with *trim45* MOs into a zebrafish embryo at one cell stage. As shown in Figure 3(D and D’), overexpression of *trim45* completely rescued the morphological defects in the midbrain and eye of *trim45* morphants in terms of the two structures and the level of *olig2* transcripts. It is thus most probable that Trim45 is essential to proper development of the midbrain and eyes in zebrafish embryogenesis.

### *Trim45* governs development of the diencephalon and eye but not of the telencephalon and midbrain–hindbrain boundary (MHB)

**Figure 4.**
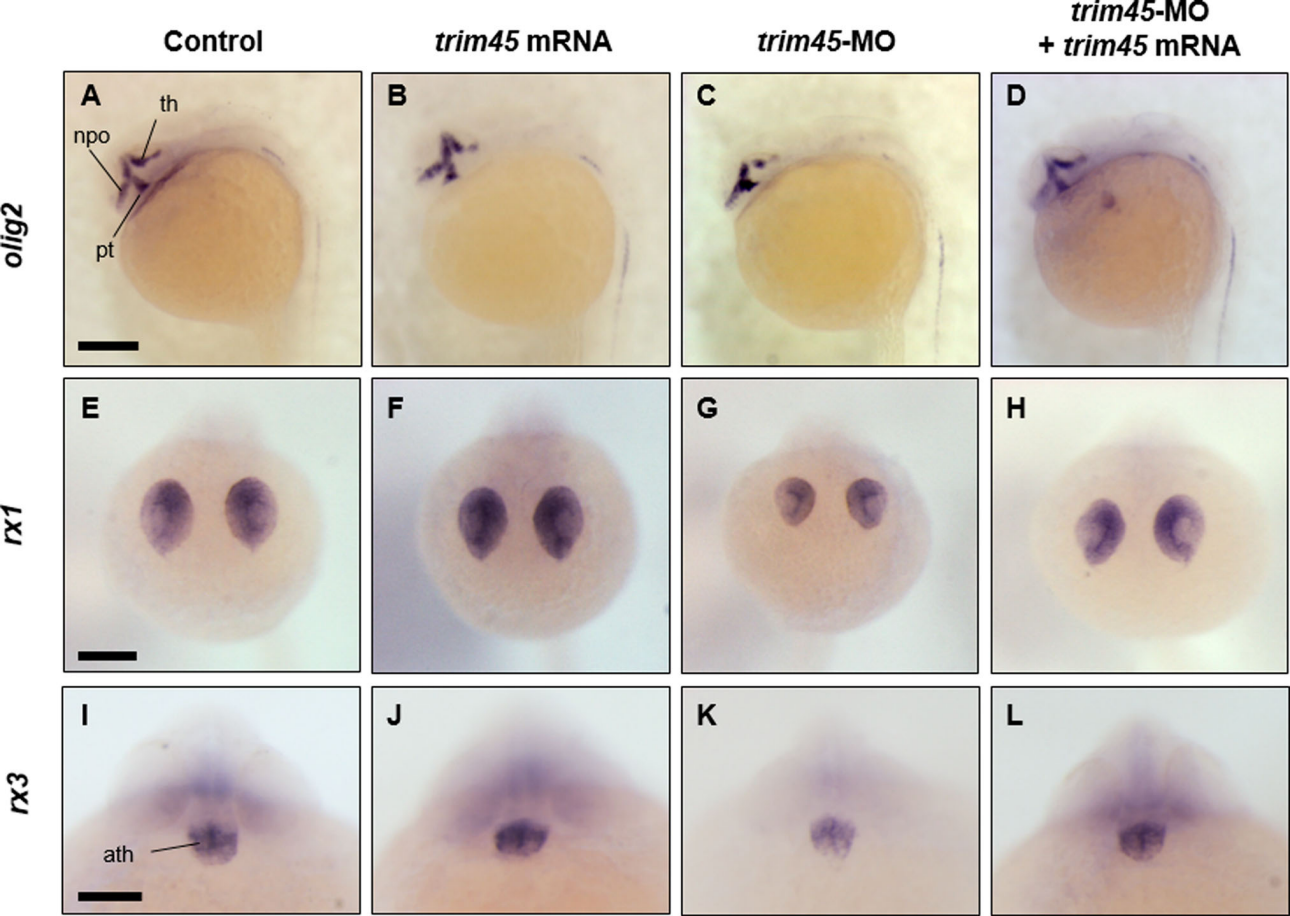
Spatio-temporal expression of *olig2*, *rx1* and *rx3*, markers for diencephalon, retina, and optic primordia, in zebrafish embryos at 24 hpf. WISH was carried out with the following markers: *olig2, rx1*, and *rx3* on each embryo (Figure 3 embryos were used). Overexpression of *trim45* showed similar expression patterns with control for *olig2* (A and B), *rx1* (E and F), and *rx3* (I and J). But, in knock-down, *trim45* morphants showed compression of *olig2* expression region and decrease in the size of thalamus (C). Also, knock-down of *trim45* induced size reduction on eyes and decrease in transcript level of *rx1* (G) and *rx3* (K). Co-injection of *trim45* mRNA with *trim45*-MO rescued the morphological phenotype of *trim45* morphants (D, H and L). Lateral views – (A–D); anterior views – (E–L) th, thalamus; npo, neurosecretory preoptic area; pt, posterior tuberculum; ath, anterior hypothalamus. Scale bar – 200 μm (A–H), 100 μm (I–L).

To define molecular events associated with functions of *trim45* in developing the midbrain and eye, we analyzed spatial distribution of various brain markers, such as *olig2*, *rx1*, *rx3*, *emx1*, and *fgf8* by conducting WISH analysis (Chuang and Raymond 2001; Loosli et al. 2003; Borodovsky et al. 2009) at 24 hpf in the embryos where *trim45* was overexpressed or knocked down at one cell stage. First, the overexpression of *trim45* did not change the spatial distribution of messages transcribed from *olig2*, a marker for diencephalon (Figure 4(B)) in comparison to WT at 24 hpf (Figure 4(A)). However, knock-down of *trim45* significantly compressed structures of the thalamus, preoptic area, and posterior tuberculum, which were labeled with *olig2*-specific probe at 24 hpf (Figure 4(C)). It is thus highly possible that Trim45 contributes to proper development of the thalamus, preoptic area, and posterior tuberculum in zebrafish embryos till 24 hpf. Second, we tested the effects of overexpression and knock-down of *trim45* on transcripts from *rx1* and *rx3*, markers for the retina and optic primordia, respectively. Overexpression of *trim45* did not cause any changes in the spatial distribution of the transcripts from *rx1* and *rx3* in the retina and optic primordia, respectively in comparison to those of WT at 24 hpf (Figure 4(E, F, I, J)). In contrast, knock-down of *trim45* not only reduced the level of the transcripts from *rx1* and *rx3*, but also remarkably compressed the size of the retina and optic primordia labeled with *rx1* and *rx3* probes, respectively at 24 hpf (Figure 4(G, K)). As expected, co-injection of *trim45* mRNAs together with *trim45* MOs into embryos at one cell stage recovered normal shapes of the thalamus, retina, and optic primordia, respectively at 24 hpf (Figure 4(D, H, L)). It is clearly suggestive conceivable that Trim45 works specifically on the development of the retina and optic primordia in zebrafish embryogenesis.

**Figure 5.**
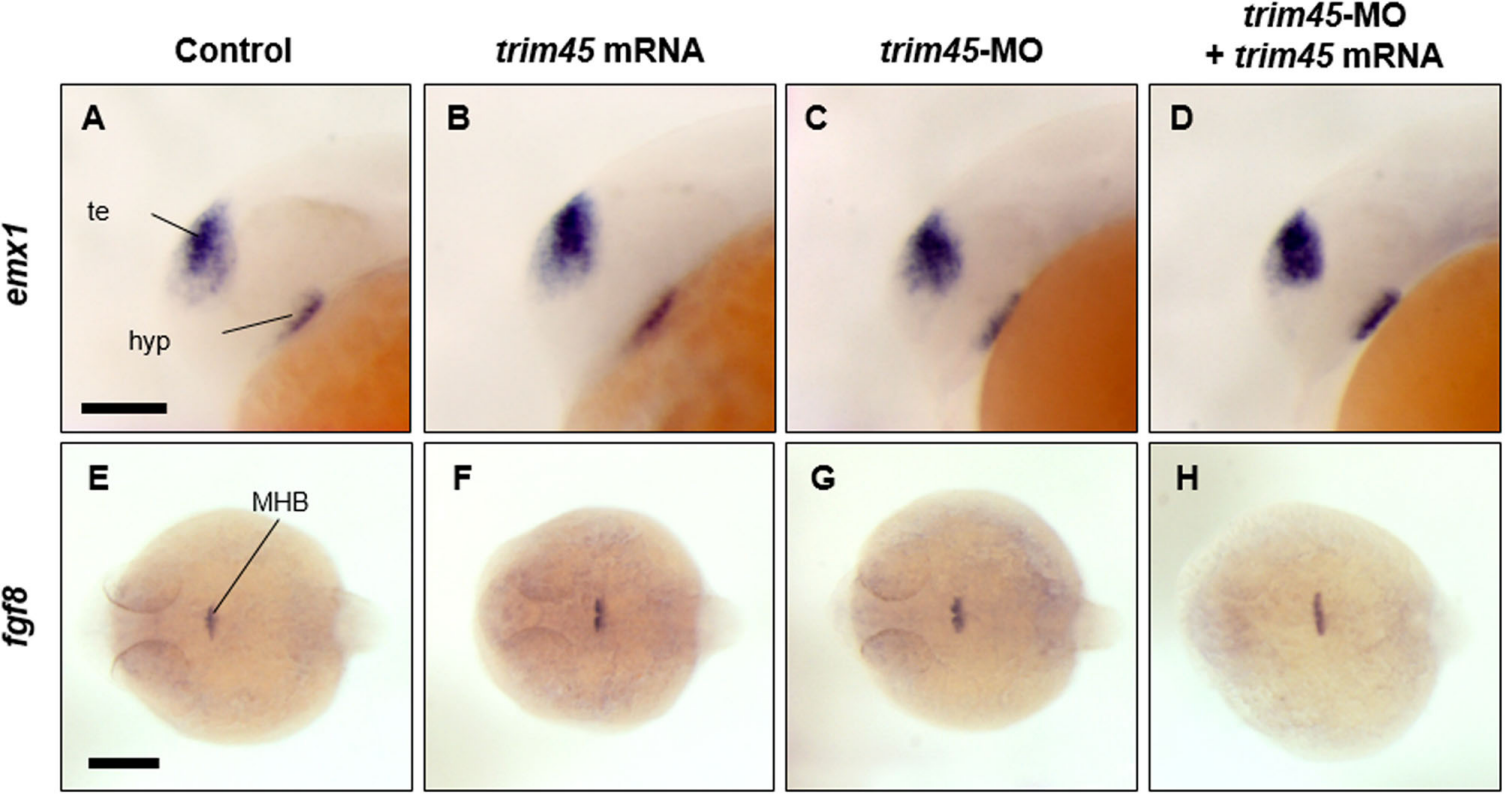
Spatio-temporal expression of *emx1* and *fgf8*, markers for telencephalon and midbrain-hindbrain boundary, in zebrafish embryos at 24 hpf. *In situ* hybridization of telencephalon marker emx1 was carried out on control (A and E), *trim45* overexpressed (B and F), *trim45* morphants (C and G), and rescued embryos (D and H) at 24 hpf. There were no significant changes on each embryo. te, telencephalon; hyp, hypothalamus; MHB, midbrain-hindbrain boundary. Scale bar – 100 μm (A–D), 200 μm (E–H).

As an attempt to examine if Trim45 exerts its functions specifically for the development of the diencephalon and eyes, we analyzed the spatial distribution of transcripts from *emx1* and *fgf8*, markers for the telencephalon and midbrain hindbrain boundary (MHB), respectively in zebrafish embryos at 24 hpf. Overexpression of *trim45* did not change spatial distribution of messages transcribed from *emx1* and *fgf8* in the telencephalon and MHB, respectively compared to those of WT at 24 hpf (Figure 5(A, B, E, F)). Furthermore, phenotypes of the telencephalon and MHB labeled with *emx1-* and *fgf8*-specific probes, respectively in *trim45* morphants were nearly identical to those of WT and the rescue groups at 24 hpf (Figure 5(C, D, G, H)). Based on all the observations from the functional studies of *trim45*, we propose that Trim45 plays essential roles in the development exclusively of the diencephalon and eye in zebrafish embryogenesis.

## Discussion

Trim45 protein is an E3 ubiquitin ligase-containing RING finger domain. Although its molecular biological functions have been studied over a decade in several cell lines, its embryological functions have not been addressed in animal models. In this study, we demonstrated that Trim45 is required specifically for the development of the diencephalon and eye but not of the telencephalon and MHB. In particular, knock-down of *trim45* changed the spatial distribution of transcripts from the three marker genes, such as *olig2*, *rx1*, and *rx3* in the diencephalon, retina, and optic primordia, respectively at 24 hpf, accompanying significant reduction of the size in the three tissues. We initially attempted to figure out time points when *trim45* is required for the development of the retina by analyzing the spatial distribution of *rx1*, a marker for retina in *trim45* morphants at 16, 18, 20, and 24 hpf. Knock-down of *trim45* significantly reduced size of the retina at all the time points (Suppl. 1) suggesting that Trim45 might be required for development of the retina as early as 16 hpf.

Because level of *trim45* transcripts appears to be critical in the development of the eyes and midbrain, we analyzed the promoter region of *trim45* (Suppl. 2)*.* When the *trim45* promoter construct expressing enhanced green fluorescent protein (EGFP) was injected into zebrafish embryos at one cell stage, EGFPs were observed in the eyes, midbrain and hindbrain region at 24–28 hpf, which overlap with expression patterns of *trim45* identified by WISH analysis. EGFPs were also detected in the muscles in the posterior part, which was also consistent with the previous studies that TRIM45 was expressed in the skeleton muscle of human embryo (Wang et al. 2004). All the observations supported that *trim45* is involved in the developmental regulation of the midbrain and eyes. Regulation of gene expression is crucial to the development of anatomy and occurs when the transcription factors (TFs) interact with specific DNA sequences called the *cis*-regulatory elements (Suryamohan and Halfon 2015). To determine which regulatory region is mostly associated with the activation of *trim45*, three different sizes of *trim45* promoter region constructs were made for promoter luciferase activity assay. Transfection of the constructs into cells for measuring luciferase activity will further quantitate transcriptional activities of the regions. Further identification of *cis-* and *trans-*acting elements regulating the spatio-temporal expression patterns of *trim45* will identify molecular elements underlying how Trim45 is associated with the development of the diencephalon and eyes.
